# Expert opinion survey on diagnostic and treatment-planning gaps in orthodontics among dental interns: Validation of the need for a MOOC-based educational intervention

**DOI:** 10.6026/973206300220829

**Published:** 2026-02-28

**Authors:** Tanusha Mahobia, Pallavi Daigavane, Piyush Khandelwal, Rudra Pratap Singh Thakur, Sonam Parakh, Neha Yadav

**Affiliations:** 1 Department of Orthodontics and Dentofacial Orthopaedics, Sharad Pawar Dental College and Hospital, Sawangi (Meghe), Wardha, Maharashtra, India; 2 Department of Orthodontics & Dentofacial Orthopedics, Chhattisgarh Dental College and Research Institute, Rajnandgaon, Chhattisgarh, India; 3 Department of Orthodontics and Dentofacial Orthopedics, Maitri College of Dentistry and Research Centre, Anjora, Durg, Chhattisgarh, India

**Keywords:** Massive Open Online Courses (MOOC), orthodontic education, diagnostic errors, dental interns, treatment planning, expert opinion

## Abstract

Accurate orthodontic diagnosis and systematic treatment planning are essential competencies for dental interns, yet inconsistencies in diagnostic skills are frequently reported. Therefore, it is of interest to expert opinion survey assessed perceived diagnostic and treatment-planning gaps among orthodontic interns and evaluated the need for a structured MOOC-based educational intervention. A validated questionnaire was administered to consultant orthodontists and responses were analyzed descriptively with thematic interpretation of qualitative inputs. Experts reported significant deficiencies in diagnostic formulation, problem listing and treatment sequencing among interns, with strong consensus on the need for structured digital training. Thus, we show the development of a focused MOOC to standardize orthodontic diagnostic education and improve clinical reasoning among interns.

## Background:

Orthodontic diagnosis requires a systematic approach involving history taking, clinical examination, radiographic interpretation and comprehensive problem listing. These steps form the foundation for effective and stable orthodontic treatment. However, undergraduate orthodontic training in many institutions remains predominantly theoretical, with limited emphasis on structured diagnostic reasoning, resulting in observable competency gaps among interns. Recent studies have reported deficiencies in diagnostic formulation and treatment planning skills among dental undergraduates and interns, particularly in transitioning from records to a definitive treatment plan [1, 6]. The rapid integration of digital technologies in dental education has enabled new instructional models, including e-learning and Massive Open Online Courses (MOOCs), which provide standardized, scalable and flexible learning opportunities [2, 7]. Evidence suggests that MOOCs and structured online modules improve knowledge retention, clinical reasoning and learner satisfaction in health professions education [3, 8]. Digital dental education has also shown effectiveness in improving diagnostic accuracy and decision-making when aligned with clinical objectives [9, 10]. Therefore, it is of interest to assess expert orthodontists' perspectives on diagnostic and treatment-planning gaps among interns and to validate the need for a structured MOOC-based educational intervention.

## Materials and Methods:

## Study design:

A descriptive cross-sectional expert-opinion survey was conducted to obtain the perspectives of consultant orthodontists on diagnostic and treatment-planning deficiencies among dental interns and to validate the need for a MOOC-based educational intervention. This design was chosen because expert-opinion studies are widely used in health-professional education to identify competency gaps, refine learning content and establish consensus on curricular priorities. The cross-sectional format allowed for efficient data collection from multiple institutions within a defined period, enabling the study to capture diverse expert experiences without the influence of temporal variability. Data were collected at a single time point using a structured paper-based questionnaire comprising closed-ended Likert-scale items and open-ended questions. This combination enhanced both the quantitative measurement of expert agreement and the qualitative exploration of underlying reasoning.

## Sample size calculation:

There is no universally accepted statistical formula for determining sample size in expert-opinion studies. Instead, literature recommends selecting a panel large enough to ensure diversity and saturation of expert perspectives. Previous health-education and dental-education studies commonly include 15-30 expert participants [1, 2, 3, 4-5]. To ensure adequate representation of both academic and clinical expertise, we targeted a minimum of 20 orthodontic consultants. This number is consistent with recommendations by Hsu & Sandford (2007) and Okoli & Pawlowski (2004), who suggest 10-30 experts for reliable consensus-based research. Thus, the final sample of 20 experts is methodologically appropriate and sufficient for robust thematic and descriptive analysis.

## Participants:

A purposive sample of 20 orthodontic consultants with ≥5 years of experience was included. Participants represented both academic and clinical backgrounds across multiple institutions in Central India.

## Questionnaire development:

A structured, paper-based questionnaire was developed specifically for this study to assess expert orthodontists' observations regarding diagnostic and treatment-planning gaps among dental interns and their perspectives on the need for a MOOC.

The questionnaire was designed after a comprehensive review of:

[1] undergraduate orthodontic curriculum guidelines,

[2] previously published studies on diagnostic errors in dentistry,

[3] expert-consensus research methods and

[4] Best-practice recommendations for educational needs assessment.

An initial item pool consisting of 28 questions was drafted. Redundant and overlapping items were removed, resulting in a final questionnaire containing 20 essential items, grouped into structured sections.

## Structure of the questionnaire:

The questionnaire consisted of seven clearly defined sections, each addressing a critical aspect of diagnostic competence and MOOC development.

## Section A: Demographics (4 items):

Collected background information to contextualize responses:

[1] years of clinical experience,

[2] type of practice (academic/clinical),

[3] institutional affiliation,

[4] Role and clinical exposure.

This ensured representation of diverse expert profiles.

## Section B: Frequency & Magnitude of Errors (3 items):

Used 5-point frequency Likert scales to quantify how often expert's encounter:

[1] Incorrect or incomplete diagnosis

[2] Inappropriate or mismatched treatment plans

[3] Negative consequences on patient satisfaction

This section established the extent of the competency gap among interns.

## Section C: Causes of Diagnostic Gaps (5 items):

Contained 5-point agreement scales to identify root causes, such as:

[1] inadequate undergraduate orthodontic teaching,

[2] insufficient clinical exposure,

[3] lack of structured diagnostic protocol,

[4] communication barriers between clinicians and orthodontists,

[5] Time constraints in general practice.

This section helped determine factors the MOOC should address.

## Section D: Impact & Consequences (4 items):

Experts rated the severity of consequences:

[1] delay in referral,

[2] loss of patient trust,

[3] increased complications,

[4] Treatment dropout.

This established the clinical relevance and public-health importance of improving diagnostic skills.

## Section E: Need for MOOC (3 items):

Assessed:

[1] agreement on MOOC necessity,

[2] Preferred format (short modules, blended learning, case bank, etc.),

[3] Recommended content areas.

This section directly guided MOOC design.

## Section F: Willingness for Delphi Validation (1 item):

Optional question asking whether experts would participate in a future case-based Delphi consensus round, supporting the rigor of further study phases.

## Section G: Open-Ended Questions (3 items):

Captured qualitative insights:

[1] most common diagnostic mistakes by interns,

[2] real-life examples of misdiagnosis,

[3] Recommendations for MOOC improvement.

These responses were crucial for thematic analysis and refining the MOOC content.

## Questionnaire format and administration:

[1] The questionnaire was paper-based to encourage participation from senior academics that may prefer offline tools.

[2] Items used simple, non-ambiguous language to improve clarity.

[3] The questionnaire required 8-12 minutes to complete.

[4] All responses were anonymous to reduce social desirability bias.

[5] Each participant received the instrument along with a written informed consent form.

## Content validation:

## The questionnaire underwent expert validation in the following stages:

## Face and content validation:

Two senior orthodontists reviewed the questionnaire for relevance, clarity and completeness.

## Pilot testing:

Conduct with three consultant orthodontists.

Minor wording adjustments were made for clarity and logical flow.

## Reliability consideration:

Since this was an expert-opinion study, internal consistency scores (Cronbach alpha) were not mandatory, but items were revised to avoid redundancy.

The study design and questionnaire development were informed by previously validated models of expert opinion surveys and digital education assessment frameworks used in dental and medical education research [4, 11]. The integration of both quantitative and qualitative components aligns with recommended mixed method approaches for evaluating educational needs and curriculum gaps [12].

## Scoring and data interpretation:

[1] Quantitative sections (B-E):

Likert-scale responses were analyzed as frequencies and percentages.

Median and interquartile ranges were used for agreement items due to ordinal data.

[2] Qualitative section (G):

Open-ended responses underwent thematic content analysis:

Coding → categorizing → theme development representative quotations included in the Results section.

## Rationale for questionnaire design:

## This structured approach ensured a comprehensive assessment of:

[1] the prevalence of diagnostic errors,

[2] the underlying causes,

[3] the clinical consequences,

[4] the perceived need for an educational intervention,

[5] The optimal structure for the proposed MOOC.

The questionnaire served as a valid and reliable tool for expert-driven needs assessment, forming the evidence base for MOOC development.

## Ethical considerations:

Approve by institutional ethics committee. Written informed consent was included in the printed questionnaire.

## Data analysis:

Data analysis consisted of both quantitative and qualitative components.

## Quantitative analysis:

All closed-ended responses were entered into Microsoft Excel and screened for missing or inconsistent entries. Categorical variables (diagnostic errors, treatment-planning mismatches, consequences, preferred MOOC formats, etc.) were summarized using frequency counts and percentages. Likert-scale responses (1-5) were analyzed as ordinal data; therefore, measures of median and interquartile range (IQR) were used instead of mean and standard deviation. This approach aligned with methodological recommendations for analyzing educational survey data

## Qualitative analysis:

## Responses to open-ended questions were analyzed using inductive thematic analysis. The process included:

[1] Initial reading and familiarization

[2] Coding of meaningful units

[3] Grouping codes into categories

[4] Developing overarching themes

Two independent coders analyzed the data to ensure credibility and reliability. Inter-coder differences were discussed and resolved through consensus. Themes were supported with anonymized direct quotations from experts to enhance richness and depth.

## Justification of approach:

Because this study aimed to understand expert perceptions and needs rather than test hypotheses or estimate population parameters, inferential statistics were not applied. The use of descriptive statistics combined with thematic analysis is well supported in health- profession education research and expert-assessment literature.

## Results:

Half of the participants had 11-20 years of professional experience, while nearly one-third had more than 20 years of experience and the majority was engaged in both academic and clinical practice. A high proportion of experts reported frequent issues related to incorrect or incomplete diagnoses, inappropriate treatment planning and a negative impact on patient confidence. Commonly observed deficiencies included misinterpretation of skeletal versus dental discrepancies, inadequate use of intraoral and extraoral examinations and poor photographic documentation. The most strongly identified contributing factors were insufficient undergraduate orthodontic teaching, inadequate clinical exposure, lack of structured diagnostic frameworks and suboptimal communication training, findings that are consistent with global concerns regarding deficiencies in dental diagnostic education. Experts rated delays in treatment initiation, loss of patient trust with associated patient burnout and treatment-related complications as highly important consequences of diagnostic errors. Several participants emphasized that incorrect advice provided at an early stage disrupts patient expectations and reduces treatment compliance. All experts supported the proposed massive open online course initiative, with a strong preference for short- duration modules and case-based learning approaches. Core components emphasized as essential included systematic clinical examination, basic clinical photography, impression making or digital scanning, case classification systems, clearly defined treatment objectives and planning, referral guidelines, step-wise case discussions with logical reasoning and foundational knowledge of orthodontic appliances and relevant surgical procedures. Qualitative thematic analysis revealed prominent curriculum-related knowledge gaps, a strong need for practical diagnostic frameworks such as checklists and simplified algorithms and significant challenges in patient communication, particularly among interns, leading to dissatisfaction.

## Discussion:

The findings of the present study highlight significant deficiencies in orthodontic diagnostic reasoning and treatment planning among dental interns, as perceived by experienced orthodontists. Similar competency gaps have been reported in recent studies evaluating undergraduate dental education outcomes, particularly in applying theoretical knowledge to clinical decision-making [6, 9]. Experts in the current study emphasized deficiencies in problem listing, case analysis and sequencing of orthodontic treatment. These observations are consistent with earlier reports suggesting that inadequate structured diagnostic training limits clinical confidence and treatment accuracy among interns [10]. The strong expert consensus supporting the development of a MOOC aligns with growing evidence that digital learning platforms enhance accessibility, standardization and learner engagement in dental education [1, 7 and 11]. MOOCs offer flexibility and repeated exposure to complex diagnostic concepts, which are often constrained by limited clinical time in conventional teaching models [2, 8]. Recent orthodontic education studies have demonstrated that online and blended learning approaches can effectively supplement chairside teaching and improve diagnostic comprehension when designed with case-based and algorithm-driven content [10]. The present study further strengthens this evidence by validating MOOC requirements through expert opinion rather than learner perception alone. Thus, the study supports the integration of structured MOOC-based orthodontic diagnostic training as a complementary strategy to conventional clinical education, particularly in resource-constrained academic settings [3].

## Conclusion:

The study validates the need for a structured MOOC to address critical gaps in orthodontic diagnosis and treatment planning among dental interns. Expert consensus supports digital education as an effective adjunct for improving clinical reasoning, diagnostic accuracy and standardization of orthodontic training.

## Advancement to knowledge:

This study provides expert-validated evidence of critical diagnostic and treatment-planning gaps among orthodontic interns. It identifies specific content domains required for structured MOOC-based orthodontic education. The findings support digital learning as a scalable strategy to standardize orthodontic diagnostic training in resource-limited settings.
